# Associations of food insecurity with geriatric syndromes in the economically deprived community-dwelling Chinese older adults: the mediating role of malnutrition risk

**DOI:** 10.1007/s41999-025-01337-2

**Published:** 2025-10-29

**Authors:** Ka Yu Kwan, Mandy Ho, Pui Hing Chau, Chee Hon Chan, Raymond Lap Ming Tang, Paul Siu Fai Yip

**Affiliations:** 1 School of Nursing, LKS Faculty of Medicine, The University of Hong Kong, Hong Kong Special Administrative Region, China; 2 Hong Kong Jockey Club Centre for Suicide Research and Prevention, The University of Hong Kong, Hong Kong Special Administrative Region, China; 3 School of Governance and Policy Science, The Chinese University of Hong Kong, Hong Kong Special Administrative Region, China; 4 Department of Social Work and Social Administration, The University of Hong Kong, Hong Kong Special Administrative Region, China

**Keywords:** Food insecurity, Geriatric syndrome, Malnutrition, Mediation, Older adults

## Abstract

**Purpose:**

Food insecurity is a vital social determinant of health. Among the economically deprived community-dwelling Chinese older adults, we examined the associations between levels of food insecurity and (1) various types and (2) the number of geriatric syndromes, and investigated the mediating role of malnutrition risk in these relationships.

**Methods:**

A cross-sectional study was conducted. 281 older adults aged ≥ 65 years with household income < 75% of the population median and participating in a food assistance program evaluation study in Hong Kong were included. Secondary data on food insecurity, malnutrition risk, possible sarcopenia, frailty, falls, and visual and hearing impairment were analyzed.

**Results:**

The mean (SD) age was 75.5 (7.1) years and 80.4% were female. The mean (SD) food insecurity score was 0.79 (1.56). The majority (88.0%) had at least one geriatric syndrome. Logistic regressions showed that every one-point increase in the food insecurity score was significantly associated with a higher malnutrition risk [adjusted Odds Ratio (aOR): 1.94, 95% CI 1.48–2.55], possible sarcopenia (aOR: 1.41, 95% CI 1.15–1.74), falls (aOR: 1.33, 95% CI 1.11–1.61), and frailty (aOR: 1.26, 95% CI 1.02–1.56), and a 43% greater risk of having one additional geriatric syndrome (aOR: 1.43, 95% CI 1.23–1.66). The proportions of association mediated by malnutrition risk were 48.6% for possible sarcopenia, 50.0% for falls, 91.3% for frailty, and 90.5% for the number of geriatric syndromes.

**Conclusion:**

Food insecurity was associated with geriatric syndromes, and malnutrition risk mediated these relationships. Tackling food insecurity may combat geriatric syndromes in the community through both its direct and indirect associations.

**Supplementary Information:**

The online version contains supplementary material available at 10.1007/s41999-025-01337-2.

## Key summary points

**Aim:**

To investigate the associations between food insecurity and geriatric syndromes, and the mediating role of malnutrition risk among economically deprived community-dwelling Chinese older adults.

**Findings:**

Food insecurity was associated with malnutrition risk, possible sarcopenia, frailty, and falls. Higher levels of food insecurity were linked to a greater number of geriatric syndromes. Furthermore, malnutrition risk mediated these relationships, with complete mediation observed specifically for the associations with frailty and the number of geriatric syndromes.

**Message:**

The findings inform policy and health services in strengthening food support services and the prevention of geriatric syndromes, hence, supporting healthy aging and the goals of sustainable development.

**Supplementary Information:**

The online version contains supplementary material available at 10.1007/s41999-025-01337-2.

## Introduction

Geriatric syndromes refer to a range of multifactorial non-disease clinical conditions that reflect cumulative impairments across multiple organ systems in older adults [1]. The Asian-Pacific Geriatric Societies provided a list of twelve conditions that can be considered geriatric syndromes, including dementia, incontinence, delirium, falls, hearing impairment, visual impairment, immobility, gait disturbances, pressure ulcers, sarcopenia, malnutrition, and frailty [2]. These conditions are often interrelated, exacerbate one another, and are associated with various adverse outcomes, such as incident chronic health conditions, lower quality of life, poorer functional ability, and greater risk of hospitalization and mortality [3–7]. This means that older adults with geriatric syndromes may require specialized care, which potentially increases both the healthcare expenditures for the individuals and the healthcare burden for the population.

Food insecurity is a social challenge for the aging population when they lack access to enough, safe, and nutritious food due to physical, social, or economic constraints [8]. It is a social determinant of negative health outcomes in older adults [9, 10], and poor health may worsen food insecurity via increased healthcare expenditures and reduced food budgets [11]. Epidemiological studies have demonstrated links between food insecurity and geriatric syndromes, such as malnutrition risk [12], sarcopenia [13], frailty [14], and falls [15]. However, research gaps remain to be addressed. First, published studies have largely been conducted in Western populations with more comprehensive pension system [16, 17], but investigations into the economically deprived Chinese older population are lacking. Particularly, in the Chinese population in Hong Kong, retired older adults mainly rely on their savings for living [18]. Second, while most studies focused on outcomes related to cognition and malnutrition risk [17], there are only scarce investigations into other geriatric syndromes. In particular, the available studies primarily examine individual geriatric syndromes, and none have explored the association between food insecurity and number of geriatric syndromes. Furthermore, malnutrition is a critical and modifiable factor in other geriatric syndromes, such as dementia, incontinence, and falls [19], but the mediating role of malnutrition or malnutrition risk in the relationships between food insecurity and other geriatric syndromes has not been well explored.

This study aimed to examine the associations between levels of food insecurity and (1) various types and (2) the number of geriatric syndromes, as well as to investigate the mediating role of malnutrition risk in these relationships among the economically deprived community-dwelling Chinese older adults.

## Methods

### Study design and study population

A secondary data analysis is based on a cross-sectional study. Data for this study were derived from the baseline evaluation study of a community-based food assistance program, which supported the economically deprived families in Hong Kong to resolve unmet food needs under the coronavirus disease (COVID-19) pandemic [20]. The evaluation study recruited a subsample of 281 participants based on the inclusion criteria: community-dwelling, Chinese, aged ≥ 65 years, currently receiving food assistance service or other social services, household income < 75% of the population median [20], and with no communication problems. About 40.2% of the 281 participants enrolled in the food assistance services, while the remaining participants were receiving other social services. Convenience and snowball samples were screened and recruited by the non-governmental organizations at 22 community centers across 12 out of 18 districts in Hong Kong territory wide between April and December 2021. Trained research assistants obtained informed consent from eligible participants and conducted face-to-face interviews and physical assessments. Participants could opt for a self-administrated mode when answering sensitive questions such as food insecurity. Upon completion, each participant received a supermarket coupon [Hong Kong Dollar, HKD50 (about USD6.4)] as an acknowledgment. Ethics approval from the Human Research Ethics Committee (HREC) of The University of Hong Kong was obtained (Reference No.: EA200233).

### Conceptual framework

The “Core Indicators of Nutritional State” model was adopted in this study as conceptual framework [10]. The model comprises determinants, indices, and consequences related to addressing public health concerns. The determinants of nutritional state include, for example, economic status, sociodemographic status, measures of capability and dependency, public sanitation/water quality, community food security, and medical conditions. The indices of nutritional state include, for example, dietary intake and food security, as well as anthropometric, biochemical, and clinical measures. The consequences of poor nutritional state include, for example, hunger, growth problems, diseases or conditions, and impaired performance. Therefore, in this study, food insecurity, which is one of those indices of nutritional state in the model, was considered the cause of geriatric syndromes and treated as an independent variable. Each dichotomized geriatric syndrome and the number of geriatric syndromes, which represent consequences of poor nutritional state by reflecting diseases or conditions, were treated as dependent variables.

Furthermore, in the model, food insecurity is considered the cause of malnutrition, and malnutrition causes impaired health conditions [10]. In geriatric research, malnutrition has often been reported as an adverse health outcome from social determinants, such as food insecurity [21]. Meanwhile, malnutrition has also been reported to induce other adverse health outcomes, such as geriatric syndromes [19]. Food insecurity leads to insufficient or imbalanced intake of energy, macronutrients, and micronutrients [22, 23], which results in a state of malnutrition, characterized by indicators such as severe weight loss and reduction of serum albumin [24]. Malnutrition impairs muscle mass and strength, immune function, and overall physiological resilience [25], which precipitates or worsens geriatric syndromes, such as sarcopenia, frailty, and falls [26]. This pathway highlights the mechanistic role of malnutrition between food insecurity and geriatric syndromes. Hence, malnutrition was selected as the mediator in this study. As malnutrition could not be diagnosed in this study, we investigated malnutrition risk instead. Although other indices of nutritional state from the conceptual model can potentially serve as mediators [10], we focused on malnutrition risk as it has the above evidence to support its mediation mechanism.

### Food insecurity

Food insecurity was measured by the 6-item Chinese version of the Household Food Security Survey Module (HFSSM) (3-month reference period), which was modified from the original HFSSM for the United States population [27]. The modification is comprised of three steps. First, the 18-item Chinese version of the HFSSM [28], was used as the basis for the modification. Second, corresponding items from the 6-item English version of the HFSSM [29], were then selected from the 18-item Chinese version of HFSSM. Given the fluctuating nature of food insecurity, particularly among economically deprived populations facing financial instability, a 3-month timeframe may offer a balance between minimizing recall bias, capturing shorter term patterns and excluding transient changes. Hence, in the third step, the reference period was changed from “12” to “3 months”, and the options for the frequency-of-occurrence item were changed from “months” to “weeks”. It demonstrated acceptable validity and reliability for measuring household food insecurity levels in the economically deprived community-dwelling Chinese population [30]. The item calibration hierarchy and goodness-of-fit of the items for the modified scale was comparable to that of the original one [31]. The internal consistency had fair-to-good internal consistency (Cronbach's alpha = 0.88 and Rasch person reliability = 0.64) and excellent test–retest reliability (intraclass correlation coefficient = 0.96). Details of the validation findings were presented elsewhere [30]. The modified scale is presented in Supplementary Table 1. The total score ranged from 0 to 6, with a higher score indicating a higher level of food insecurity. If one or two items were missing, they were imputed based on the item-severity order of the scale. However, cases missing more than three items were excluded from the analyses [27]. In the main analysis, the score was used as a continuous variable. In sensitivity analysis, a categorical variable defined by the cut-off point of the modified scale was used (0–3 affirmative responses were classified as “normal to low levels of food insecurity”; 4–6 affirmative responses were classified as “high levels of food insecurity”).

### Geriatric syndromes

Malnutrition risk was screened using the Mini Nutritional Assessment-Short Form (MNA®-SF) [32, 33]. It is a robust, global, and simple tool for community-dwelling older adults [24]. The tool consists of 6 items covering unintentional food intake reduction, unintentional weight loss, mobility, psychological stress or acute disease, neuropsychological problems, and body mass index (BMI) or calf circumference. The total score ranged from 0 to 14, with a cut-off score of 11 or below indicating at risk or malnourished.

Possible sarcopenia was defined as those screened to have sarcopenia symptoms and with low muscle strength and/or low physical performance, according to the Asian Working Group for Sarcopenia (AWGS) 2019 diagnostic algorithm [34]. Sarcopenia symptoms were indicated by a score of 11 or above using the validated 5-item SARC-CalF questionnaire [35]. The scale covered strength of muscle, assistance in walking, rise from a chair, climb stairs, falls, and calf circumference. The total score ranged from 0 to 20. Participants scoring ≥ 11 on the SARC-CalF were assessed for muscle strength and physical performance. Muscle strength was measured using a digital spring-type dynamometer (model: TKK5401) in a standing position with full elbow extension. The maximum reading of the four trials (two from each hand) was used for analysis. According to the AWGS 2019, both spring-type and hydraulic-type dynamometers are recommended to measure muscle strength in the process of sarcopenia identification [34]. The dynamometer (TKK models) was reported to have good reliability and validity [36]. Physical performance was measured by the 5-time chair stand test, which required timing the performance of sit-to-stand in five repetitions at the fastest speed and with arms crossed over the chest. Participants with low muscle strength (i.e., male < 28 kg or female < 18 kg in the handgrip strength test) and/or low physical performance (i.e., ≥ 12 s in the 5-time chair stand test) were considered as having possible sarcopenia [34].

Frailty was screened using the validated 5-item FRAIL questionnaire [37, 38], which consists of fatigue, resistance, ambulation, illness, and loss of weight. This brief screening tool is feasible to detect frailty in the Chinese community [39]. The total score ranged from 0 to 5, with a cut-off score of 1 or above indicating pre-frail or frail.

Falls were identified if the participants had a self-reported history of at least one fall (both injurious and non-injurious) in the past 12 months.

Visual impairment was identified by self-reported visual problems that hinder the judgment of distance when going down the stairs despite the use of visual aids.

Hearing impairment was identified by self-reported hearing problems that hinder daily communication despite the use of hearing aids.

### Sociodemographic, health condition, and lifestyle

Based on literature, age, sex, educational level, marital status, living alone, monthly household income, residential areas, multimorbidity, self-rated health, weight status, and physical activity level were included as confounders [1, 40]. Household income was dichotomized based on the median monthly household income of the low-income households led by older adults in Hong Kong, with a cut-off at HKD4200 (about USD538.5) [41]. Multimorbidity was defined as having two or more chronic diseases [42]. Weight status (i.e., BMI) was classified into underweight (< 18.5 kg/m^2^), normal (18.5–22.9 kg/m^2^), overweight (23.0–24.9 kg/m^2^), and obese (≥ 25.0 kg/m^2^) based on the Asian-specific cutoffs [43]. Physical activity level was classified into inactive, minimally active, and active according to the scoring algorithm of the International Physical Activity Questionnaire-Short Form [44].

### Statistical analysis

Descriptive statistics were used to describe the sample characteristics. Participants with missing data were excluded from the respective analyses. The proportions of each geriatric syndrome and the number of geriatric syndromes were estimated with 95% confidence intervals (CIs). Binary logistic regression was used to investigate the associations between food insecurity scores and each dichotomized geriatric syndrome. Ordinal logistic regression was used to investigate the association between food insecurity scores and the number of geriatric syndromes. Both unadjusted and adjusted odds ratios (aORs) with 95% CI were estimated. The adjustment included the sociodemographic, health condition, and lifestyle variables. Multicollinearity was assessed and indicated by a variance inflation factor (VIF) of less than 5 [45]. Proportional odds assumption was examined using the test of parallel lines. Goodness-of-fit for binary and ordinal logistic regression models was examined using Hosmer–Lemeshow test and Chi-square test, respectively.

**Fig. 1:**
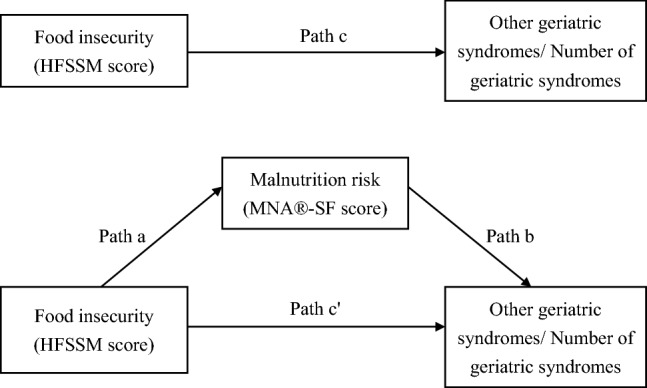
Proposed mediation model for food insecurity, malnutrition risk, and other geriatric syndromes (binary outcome) or the number of geriatric syndromes (continuous outcome). The point estimate from path c quantifies the total effect of food insecurity on other geriatric syndromes or the number of geriatric syndromes. The multiplication of the point estimates from path a and path b provides the indirect effect of malnutrition risk on the relationship between food insecurity and other geriatric syndromes or the number of geriatric syndromes. The point estimate from path c' quantifies the direct effect of food insecurity on other geriatric syndromes or the number of geriatric syndromes after adjusting for malnutrition risk. Abbreviations: HFSSM, Household Food Security Survey Module; MNA®-SF, Mini Nutritional Assessment-Short Form

Figure 1 shows the mediation analysis model. The total effect was estimated using the logistic or linear regression of the food insecurity scores on the outcomes, which were the dichotomized geriatric syndromes or the number of geriatric syndromes (Path c). The direct effect was estimated by regressing the outcomes on food insecurity scores (Path c'), adjusting for malnutrition. The regression coefficient of malnutrition in the same regression (Path b), multiplied by the regression coefficient from the regression of malnutrition on the food insecurity scores (Path a), gave the indirect effect (mediation). Bias-corrected bootstrapping with 5000 bootstrap samples was used to generate 95% CIs for the indirect effect, with significance indicated if the 95% CI did not include zero. The mediated proportion was calculated as: [indirect effect / (direct effect + indirect effect) × 100%]. Complete mediation was indicated when the significant relationship between the food insecurity scores and the outcomes became insignificant after adjusting for the mediator, and the mediated proportion recommended to be greater than 80% [46].

In addition, to examine the robustness of treating the food insecurity score as a continuous variable, sensitivity analyses were performed by categorizing food insecurity using the cut-off of the modified scale described in "Food insecurity" section.

All statistical analyses were performed using SPSS version 28.0, and a 5% level of significance was adopted. The PROCESS macro version 4.2 for SPSS (model 4) [47] was used for mediation analysis.

## Results

Table 1 shows the characteristics of the 281 participants. The mean (standard deviation [SD]) age of the participants was 75.5 (7.1) years. The majority (80.4%) of the participants were female, 41.3% were living alone, and 52.3% had multimorbidity. The mean food insecurity score was 0.79 (95% CI 0.61–0.97). Among the six studied geriatric syndromes, frailty was the most prevalent one (62.3%, 95% CI 56.6–67.9%), followed by malnutrition risk (46.4%, 95% CI 40.6–52.3%), visual impairment (33.1%, 95% CI 27.6–38.6%), hearing impairment (24.6%, 95% CI 19.5–29.6%), falls (23.5%, 95% CI 18.5–28.4%), and possible sarcopenia (21.4%, 95% CI 16.5–26.2%). Only 12.0% (95% CI 8.1–15.8%) of the participants had no geriatric syndromes, 26.8% (95% CI 21.6–32.0%) of the participants had one geriatric syndrome, and 61.2% (95% CI 55.5–67.0%) had more than one geriatric syndrome. On average, each participant experienced 2.06 (SD = 1.30) geriatric syndromes.

**Table 1:** Characteristics of the participants analyzed in this study (*n* = 281)

Characteristics	*n* (%)	Mean (SD)
Age, years (range: 65–94)		75.5 (7.1)
Food insecurity score		0.79 (1.56)
Sex (female)	226 (80.4)	
Education		
Primary school or below	188 (66.9)	
Secondary school or above	93 (33.1)	
Marital status		
Single, divorced, separated, or widowed	159 (56.6)	
Married or cohabitated	122 (43.4)	
Living alone	116 (41.3)	
Below median monthly household income^a^	135 (48.0)	
Residential area		
Hong Kong Island	46 (16.4)	
Kowloon	188 (66.9)	
New Territories	47 (16.7)	
Multimorbidity	147 (52.3)	
Self-rated health		
Poor or fair	202 (71.9)	
Good or above	79 (28.1)	
Weight status (*n* = 275^b^)		
Underweight (BMI < 18.5 kg/m^2^)	16 (5.8)	
Normal (BMI 18.5–22.9 kg/m^2^)	82 (29.8)	
Overweight (BMI 23.0–24.9 kg/m^2^)	60 (21.8)	
Obese (BMI ≥ 25.0 kg/m^2^)	117 (42.5)	
Physical activity level		
Inactive	23 (8.2)	
Minimally active^c^	257 (91.5)	
Active^c^	1 (0.4)	
Malnutrition risk (At risk or malnourished) (*n* = 280^d^)	130 (46.4)	
Possible sarcopenia (Yes) (*n* = 276^d^)	59 (21.4)	
Frailty (Pre-frail or frail)	175 (62.3)	
Falls (At least one fall in the past 12 months)	66 (23.5)	
Visual impairment (Yes)	93 (33.1)	
Hearing impairment (Yes)	69 (24.6)	
Number of geriatric syndromes (*n* = 276)		
0	33 (12.0)	
1	74 (26.8)	
2	62 (22.5)	
3	57 (20.7)	
4	50 (18.1)	

Abbreviation: BMI, body mass index; SD, standard deviation

^a^Based on the median monthly household income of the low-income households led by older adults in Hong Kong (i.e., HKD4200≈USD538.5) [41]

^b^Missing data in weight status due to health condition limitations (*n* = 6)

^c^In subsequent analyses, “active” and “minimally active” were grouped as one category due to small number of participants in “active” category

^d^Missing physical measurements due to health condition limitations (e.g., body weight, standing height, calf circumference, or 5-time sit-stand test) for scoring of malnutrition risk (*n* = 1) and possible sarcopenia (*n* = 5)

Both the unadjusted and adjusted ORs of food insecurity on geriatric syndrome estimated from the logistic regression models were consistent (Table 2). Controlling for confounders, every one unit increase in the food insecurity score was significantly associated with an increase in the likelihood of being at risk or malnourished by 94% (aOR: 1.94, 95% CI 1.48–2.55, *p* < 0.001), having possible sarcopenia by 41% (aOR: 1.41, 95% CI 1.15–1.74, *p* = 0.001), being pre-frail or frail by 26% (aOR: 1.26, 95% CI 1.02–1.56, *p* = 0.033), and having at least one fall in the past year by 33% (aOR: 1.33, 95% CI 1.11–1.61, *p* = 0.003). However, the associations for visual impairment (*p* = 0.098) and hearing impairment (*p* = 0.522) were not significant. Meanwhile, the adjusted ordinal logistic regression showed that a unit increase in the food insecurity score was significantly associated with a 43% higher likelihood of having an additional geriatric syndrome (aOR: 1.43, 95% CI 1.23–1.66, *p* < 0.001). Multicollinearity was not found (VIFs < 2), and the proportional odds assumption was not violated (*p* = 0.202 and 0.948 for unadjusted and adjusted models, respectively). Model fit was supported by Hosmer–Lemeshow tests and Chi-square tests (all *p* values > 0.05).

**Table 2:** The strength of associations of food insecurity on geriatric syndromes based on binary and ordinal logistic regression models

Geriatric syndromes^a^	Unadjusted models				Adjusted models			
	*n*	OR	(95% CI)	*p*-value	*n*	aOR^b^	(95% CI)	*p*-value
Malnutrition risk	280	1.87***	(1.47 to 2.37)	< 0.001	275	1.94***	(1.48 to 2.55)	< 0.001
Possible sarcopenia	276	1.31**	(1.12 to 1.54)	0.001	271	1.41**	(1.15 to 1.74)	0.001
Frailty	281	1.26*	(1.05 to 1.52)	0.014	275	1.26*	(1.02 to 1.56)	0.033
Falls	281	1.27**	(1.08 to 1.49)	0.003	275	1.33**	(1.11 to 1.61)	0.003
Hearing impairment	281	1.07	(0.90 to 1.26)	0.463	275	1.06	(0.88 to 1.28)	0.522
Visual impairment	281	0.89	(0.75 to 1.06)	0.200	275	0.85	(0.70 to 1.03)	0.098
Number of geriatric syndromes^c^	276	1.38***	(1.21 to 1.58)	< 0.001	271	1.43***	(1.23 to 1.66)	< 0.001

Abbreviation: aOR, adjusted odds ratio; CI, confidence interval; OR, odds ratio

^*^p < 0.05; **p < 0.01; ***p < 0.001

^a^Individual geriatric syndromes were analyzed separately by binary logistic regression models, and the number of geriatric syndromes was analyzed by ordinal logistic regression model

^b^Adjusted for age, sex, education, marital status, living alone, monthly household income, residential areas, multimorbidity, self-rated health, weight status, and physical activity level

^c^The number of geriatric syndromes was counted from the presence of malnutrition risk, possible sarcopenia, frailty, falls, visual impairment, and hearing impairment

Table 3 presents the results of the mediation analyses between food insecurity, malnutrition risk, and other geriatric syndromes. The total effects of food insecurity scores on possible sarcopenia, frailty, falls, and the number of geriatric syndromes were all significant. After adjusting for the malnutrition risk score, the direct effects of food insecurity scores on all other geriatric syndromes were no longer significant (possible sarcopenia, *p* = 0.095; frailty, *p* = 0.840; falls, *p* = 0.148; number of geriatric syndromes, *p* = 0.572). The indirect effects in all the mediation models were significant (possible sarcopenia, coefficient: 0.18, 95% CI 0.06–0.31; frailty, coefficient: 0.21, 95% CI 0.11–0.32; falls, coefficient: 0.15, 95% CI 0.05–0.26; number of geriatric syndromes, coefficient: 0.19, 95% CI 0.14–0.24). The mediating effect of malnutrition explained 48.6% of the relationship between food insecurity and possible sarcopenia, 50.0% with falls, 91.3% with frailty, and 90.5% with the number of geriatric syndromes.

**Table 3:** Results of the mediation analysis for food insecurity, malnutrition risk, and other geriatric syndromes

Other geriatric syndromes	Path c: food insecurity and other geriatric syndromes (Total effect)		Path a: food insecurity and malnutrition risk		Path b: malnutrition risk and other geriatric syndromes		Path cʹ: food insecurity and other geriatric syndromes (Direct effect)		Path a × Path b (Indirect effect)		Mediated proportion
	B^a^	(95% CI)	B^a,b^	(95% CI)	B^a^	(95% CI)	B^a^	(95% CI)	B^a^	(95% CI)^c^	%
			− 0.52***	(− 0.67 to − 0.37)							
Possible sarcopenia^d^ (*n* = 271; binary outcome)	0.34**	(0.14 to 0.55)			− 0.34***	(− 0.54 to − 0.15)	0.19	(− 0.03 to 0.42)	0.18^#^	(0.06 to 0.31)	48.6
Frailty^d^ (*n* = 275; binary outcome)	0.23*	(0.02 to 0.45)			− 0.41***	(− 0.59 to − 0.23)	0.02	(− 0.21 to 0.26)	0.21^#^	(0.11 to 0.32)	91.3
Falls^d^ (*n* = 275; binary outcome)	0.29**	(0.10 to 0.48)			− 0.30***	(− 0.47 to − 0.12)	0.15	(− 0.05 to 0.35)	0.15^#^	(0.05 to 0.26)	50.0
Number of geriatric syndromes^e^ (*n* = 271; continuous outcome)	0.21***	(0.12 to 0.30)			− 0.36***	(− 0.42 to −0.30)	0.02	(− 0.06 to 0.10)	0.19^#^	(0.14 to 0.24)	90.5

Abbreviation: B, regression coefficient; CI, confidence interval

^*^p < 0.05; **p < 0.01; ***p < 0.001

^#^Statistical significance was based on the 95% CI

^a^Regression coefficients adjusted for age, sex, education, marital status, living alone, monthly household income, residential areas, multimorbidity, self-rated health, weight status, and physical activity level

^b^The sample size of path a depends on the mediation models being tested. Despite the sample size varied across the models, the results of path a were nearly identical

^c^Based on bias-corrected bootstrap with 5000 bootstrap samples

^d^Path c and path c' were analyzed based on binary logistic regressions, and the coefficients were presented as a log-odds metric

^e^The number of geriatric syndromes was counted from the presence of malnutrition risk, possible sarcopenia, frailty, falls, visual impairment, and hearing impairment

The findings from the sensitivity analysis are presented in Supplementary Tables 2 and 3. Significant associations between food insecurity with all geriatric syndromes, and the insignificant associations with hearing or visual impairments, were consistently reported. The mediation role of malnutrition risk was also consistent with the main analysis, despite the mediated proportions being smaller in magnitude.

## Discussion

To the best of our knowledge, this is the first study to include multiple geriatric syndromes and the number of geriatric syndromes to examine their associations with food insecurity. Food insecurity was significantly associated with higher malnutrition risk, possible sarcopenia, falls, frailty, and having more geriatric syndromes. The mediating role of malnutrition risk in the associations of food insecurity with possible sarcopenia, falls, frailty, and the number of geriatric syndromes was also reported.

We estimated that each unit increase in the food insecurity score was associated with an aOR ranging from 1.26 to 1.94 for various geriatric syndromes. Most prior cross-sectional studies investigated food insecurity as a categorical variable, reporting an aOR ranging from 1.73 to 4.45 for malnutrition risk [12, 48–52]; an aOR of 1.51 for probable sarcopenia [53]; an aOR ranging from 1.31 to 4.69 for frailty [14, 54–56]; an aOR ranging from 1.69 to 1.95 for falls [15, 57]; an aOR ranging from 0.76 to 2.62 for visual impairment [58–61]; and an aOR ranging from 1.35 to 1.83 for hearing impairment [61, 62]. In recent years, some studies have investigated the longitudinal associations between food insecurity and geriatric syndromes. For example, one study based on nationally representative data from Mexico found that moderate and severe food insecurity was linked to higher rates of sarcopenia and severe sarcopenia over a 12-year follow-up period [63]. Similarly, research using national data from the United States revealed that food insecurity increased the risk of frailty and falls over an 8-year span [64]. While these increased risks were largely consistent, the insignificant associations for visual and hearing impairments reported in our study may be partly due to different inclusion criteria, recruitment procedures, measurement methods, and analytical techniques across studies. Nevertheless, our study contributed to the literature by analyzing these relationships as a quantitative variable, which allows a better description of the associations between varying levels of food insecurity with geriatric syndromes.

Besides, using the number of geriatric syndromes as an outcome, our findings demonstrated a positive relationship. A higher level of food insecurity represents a higher level of stressor [27], which may negatively impact on body functions [65, 66]. A recent Canadian study demonstrated that the relationship between higher perceived stress and poorer cognitive function was significant with low adherence to the Mediterranean diet [67]. This evidence supports that the combined psychological and nutritional disturbances induced by high levels of food insecurity may harm physiological reserves to a greater extent. Therefore, resolving food insecurity is a critical step to minimizing health decline.

Our results showed that food insecurity was associated with similar risks of malnutrition, possible sarcopenia, frailty, and falls. One possible explanation is that these four geriatric syndromes are closely interrelated to form a vicious cycle. It has been suggested that sarcopenia mediates the relationship between malnutrition and frailty [26, 68], and falls often occur subsequent to frailty [69]. Our mediation analyses further revealed that malnutrition risk mediates the association between food insecurity and other geriatric syndromes. More specifically, complete mediation was found for the relationships with frailty and the number of geriatric syndromes. These findings highlight the importance of interventions, such as food support services, which may help reduce malnutrition risk and, indirectly, mitigate the development of other geriatric syndromes. Given that malnutrition risk emerged as a key mediator, older adults experiencing both food insecurity and high malnutrition risk may be given higher priority to receiving interventions. More importantly, dietitians should regularly review the quality of food assistance services and closely monitor the diets of service users to ensure that malnutrition is well targeted.

This study comprised of a number of strengths. First, by including six geriatric syndromes within a single context, the study allowed for the comparison of the strength of associations and enabled the analysis of the number of geriatric syndromes as a quantitative outcome. Moreover, most of these measures involved quantifiable or observable items (e.g., BMI value, weight loss, grip strength, etc.) to maintain objectivity. Second, validated and context-specific tools were used to measure food insecurity and geriatric syndromes. Nevertheless, subject to the availability of a validated objective measure of food insecurity, the analyses could be revisited. Third, the homogeneity of the studied population, restricted to an economically deprived group, could improve internal validity by reducing unmeasured potential confounders such as neighborhood environment. On the other hand, this study consists of several limitations. First, the cross-sectional study design limited the inference of temporal precedence of food insecurity on geriatric syndromes, and did not necessarily reflect causality in the mediation models. Second, being a secondary data analysis, some geriatric syndromes, such as incontinence, delirium, and dementia, were not available in the evaluation study of the community-based food assistance program and thus could not be explored. Third, the current gold standard to confirm malnutrition is based on the Global Leadership Initiative on Malnutrition (GLIM) criteria, but it was not available in our study; hence, MNA®-SF, which is considered as an acceptable proxy [32], was adopted. Fourth, the number of geriatric syndromes was treated as a continuous outcome in the mediation analysis, since the SPSS PROCESS macro-analyzes ordinal outcome using ordinary least-squares regression. Fifth, the focus on a single region in China may limit generalizability, as Hong Kong is a highly urbanized city that may not represent other less-developed regions in China. Besides, this study focused on the economically deprived individuals, who may differ from those who are not economically deprived. The mean food insecurity score of 0.79 might imply that the studied population was not having serious food insecurity. This may be limited by a mix of users and non-users of food assistance services in the sample. Moreover, as this study focused on older adults only, our previous study showed that older adults were less likely experienced food insecurity when compared with younger adults [30]. Finally, the use of a convenience sample might introduce selection bias, as older adults who were too frail to visit the community centers were unlikely to be included.

In future research, we recommend examining the longitudinal association of change of food insecurity on geriatric syndromes. Also, multiple mediators and moderators underlying the associations between food insecurity and geriatric syndromes should be explored. These studies would provide additional evidence for developing more appropriate interventions to minimize the progressing impacts of food insecurity on the older population. Furthermore, to enhance generalizability, there is a need for multi-center validation in China. In terms of practice, policymakers should address both food insecurity and geriatric syndromes, particularly malnutrition.

In conclusion, this study demonstrates positive relationships between food insecurity and geriatric syndromes, with these relationships being mediated by malnutrition risk. Tackling food insecurity may combat geriatric syndromes in the community through both its direct and indirect effects.

## Supplementary Information

Below is the link to the electronic supplementary material.

Supplementary file1 (DOCX 31 KB)

## Data Availability

Data described in the manuscript will not be made publicly available due to ethical restrictions. Aggregate data are available from the corresponding author upon reasonable request.
