# Supplementary material for: Associations of food insecurity with geriatric syndromes in the economically deprived community-dwelling Chinese older adults: the mediating role of malnutrition risk
Source: Eur Geriatr Med. 2025 Oct 29;16(6):2259–69. doi: 10.1007/s41999-025-01337-2 (PMC12743659; doi:10.1007/s41999-025-01337-2)
Supplement: Supplementary file 1 — Supplementary file1 (DOCX 31 KB) [file 41999_2025_1337_MOESM1_ESM.docx]

**Supplementary materials**

**Journal:** *European Geriatric Medicine*

**Associations of food insecurity with geriatric syndromes in the economically deprived community-dwelling Chinese older adults: The mediating role of malnutrition risk**

Ka Yu Kwan^1^ (MPhil), Mandy Ho^1^ (PhD), Pui Hing Chau^1,2,*^ (PhD), Chee Hon Chan^3^ (PhD), Raymond Lap Ming Tang^2^ (MAPPM), Paul Siu Fai Yip^2,4^ (PhD)

**Author affiliations:**

1. School of Nursing, LKS Faculty of Medicine, The University of Hong Kong, Hong Kong Special Administrative Region, China
2. Hong Kong Jockey Club Centre for Suicide Research and Prevention, The University of Hong Kong, Hong Kong Special Administrative Region, China
3. School of Governance and Policy Science, The Chinese University of Hong Kong, Hong Kong Special Administrative Region, China
4. Department of Social Work and Social Administration, The University of Hong Kong, Hong Kong Special Administrative Region, China

***Corresponding author:** P. H. Chau; Associate Professor; Address: Room 530, 5/F, Academic Building, 3 Sassoon Road, Pokfulam, Hong Kong; Email: [phchau@graduate.hku.hk](mailto:phchau@graduate.hku.hk); Telephone: +852 3917 6626

**Supplementary Table 1** The Chinese translation of the modified HFSSM.

**「家庭飲食無憂調查單元」: 六題、中文、三個月參照期之版本**

| 以下問題是關於你的家庭在過去的3個月內，即是從(___)月起，所吃的食物，以及關於你是否能夠負擔你所需要的食物。 | |
| --- | --- |
| 1. 「我/我們購買的食物不夠，而且沒有錢去買更多的食物。」在過去的3個月中，這種情況有幾經常發生在(你/你家人)身上呢? | ☐ 經常發生 |
|  | ☐ 有時候發生 |
|  | ☐ 從來沒有發生 |
|  | ☐ 不知道或拒絕回答 |
| 2. 「(我/我們)無法負擔均衡的飲食。」在過去的3個月中，這種情況有幾經常發生在(你/你家人)身上呢? | ☐ 經常發生 |
|  | ☐ 有時候發生 |
|  | ☐ 從來沒有發生 |
|  | ☐ 不知道或拒絕回答 |
| 3. 在過去的3個月中，(你/你家中的其他成年人)，有沒有因為不夠錢購買食物，而減少食物的份量，或者減少餐數？ | ☐ 有 |
|  | ☐ 沒有………跳至第5題 |
|  | ☐ 不知道……跳至第5題 |
| 4. (假如上一題的答案是「有」，則問這問題) 這種情況多久發生一次? | ☐ 幾乎每個星期 |
|  | ☐ 有些星期，但不是每個星期 |
|  | ☐ 只是1個或2個星期 |
|  | ☐ 不知道或拒絕回答 |
| 5. 在過去的3個月中，你有沒有因為不夠錢購買食物，而吃得比你該吃的少? | ☐ 有 |
|  | ☐ 沒有 |
|  | ☐ 不知道或拒絕回答 |
| 6. 在過去的 3個月中，你有沒有因為不夠錢購買食物，而感到飢餓但沒有進食? | ☐ 有 |
|  | ☐ 沒有 |
|  | ☐ 不知道或拒絕回答 |

| **Supplementary Table 2** The strength of associations of food insecurity (as categorical variable) on geriatric syndromes based on binary and ordinal logistic regression models. | | | | | | | | | | |
| --- | --- | --- | --- | --- | --- | --- | --- | --- | --- | --- |
| **Geriatric syndromes**^a^ | **Unadjusted models** | | | |  | **Adjusted models** | | | | |
|  | **n** | **OR** | **(95% CI)** | **p-value** |  | **n** | **aOR**^b^ | **(95% CI)** | **p-value** |  |
| Malnutrition risk | 280 | 18.50*** | (4.30 to 79.65) | <0.001 |  | 275 | 19.95*** | (4.22 to 94.29) | <0.001 |  |
| Possible sarcopenia | 276 | 3.21** | (1.42 to 7.23) | 0.005 |  | 271 | 5.13** | (1.77 to 14.83) | 0.003 |  |
| Frailty | 281 | 4.05* | (1.37 to 12.03) | 0.012 |  | 275 | 4.50* | (1.31 to 15.45) | 0.017 |  |
| Falls | 281 | 2.33* | (1.03 to 5.26) | 0.042 |  | 275 | 3.06* | (1.17 to 8.01) | 0.023 |  |
| Hearing impairment | 281 | 1.52 | (0.66 to 3.55) | 0.328 |  | 275 | 1.76 | (0.68 to 4.58) | 0.248 |  |
| Visual impairment | 281 | 0.65 | (0.27 to 1.58) | 0.340 |  | 275 | 0.48 | (0.18 to 1.33) | 0.159 |  |
| Number of geriatric syndromes^c^ | 276 | 4.41*** | (2.21 to 8.83) | <0.001 |  | 271 | 5.69*** | (2.57 to 12.60) | <0.001 |  |
| Abbreviation: aOR, adjusted odds ratio; CI, confidence interval; OR, odds ratio.  *p<0.05; **p<0.01; ***p<0.001.  ^a^ Individual geriatric syndromes were analyzed separately by binary logistic regression models, and the number of geriatric syndromes was analyzed by ordinal logistic regression model.  ^b^ Adjusted for age, sex, education, marital status, living alone, monthly household income, residential areas, multimorbidity, self-rated health, weight status, and physical activity level.  ^c^ The number of geriatric syndromes was counted from the presence of malnutrition risk, possible sarcopenia, frailty, falls, visual impairment, and hearing impairment. | | | | | | | | | | |

| **Supplementary Table 3** Results of the mediation analysis for food insecurity (as categorical variable), malnutrition risk, and other geriatric syndromes. | | | | | | | | | | | | | | | | | | | | | | | | | | | |
| --- | --- | --- | --- | --- | --- | --- | --- | --- | --- | --- | --- | --- | --- | --- | --- | --- | --- | --- | --- | --- | --- | --- | --- | --- | --- | --- | --- |
| **Other geriatric syndromes** |  | **Path c: Food insecurity and other geriatric syndromes (Total effect)** | | | |  | **Path a: Food insecurity and malnutrition risk** | | | |  | **Path b: Malnutrition risk and other geriatric syndromes** | | | |  | **Path c': Food insecurity and other geriatric syndromes (Direct effect)** | | | |  | **Path a x Path b (Indirect effect)** | | | |  | **Mediated proportion** |
|  |  | **B^a^** | **(95% CI)** | | |  | **B^a,b^** | **(95% CI)** | | |  | **B^a^** | **(95% CI)** | | |  | **B^a^** | **(95% CI)** | | |  | **B^a^** | **(95% CI)**^c^ | | |  | **%** |
|  |  |  |  |  |  |  | −2.24*** | (−3.05 | to | −1.42) |  |  |  |  |  |  |  |  |  |  |  |  |  |  |  |  |  |
| **Possible sarcopenia**^d^ (n=271; binary outcome) |  | 1.64** | (0.57 | to | 2.70) |  |  |  |  |  |  | −0.35*** | (−0.55 | to | −0.16) |  | 0.98 | (−0.16 | to | 2.12) |  | 0.79^#^ | (0.29 | to | 1.37) |  | 44.6 |
| **Frailty**^d^ (n=275; binary outcome) |  | 1.50* | (0.27 | to | 2.74) |  |  |  |  |  |  | −0.39*** | (−0.56 | to | −0.22) |  | 0.70 | (−0.64 | to | 2.04) |  | 0.88^#^ | (0.44 | to | 1.36) |  | 55.7 |
| **Falls**^d^  (n=275; binary outcome) |  | 1.12* | (0.16 | to | 2.08) |  |  |  |  |  |  | −0.32*** | (−0.49 | to | −0.15) |  | 0.50 | (−0.52 | to | 1.51) |  | 0.72^#^ | (0.29 | to | 1.19) |  | 59.0 |
| **Number of geriatric syndromes**^e^ (n=271; continuous outcome) |  | 1.03*** | (0.55 | to | 1.51) |  |  |  |  |  |  | −0.35*** | (−0.41 | to | −0.29) |  | 0.24 | (−0.16 | to | 0.65) |  | 0.79^#^ | (0.54 | to | 1.07) |  | 76.7 |
| Abbreviation: B, regression coefficient; CI, confidence interval.  *p<0.05; **p<0.01; ***p<0.001.  ^#^Statistical significance was based on the 95% CI.  ^a^ Regression coefficients adjusted for age, sex, education, marital status, living alone, monthly household income, residential areas, multimorbidity, self-rated health, weight status, and physical activity level.  ^b^ The sample size of path a depends on the mediation models being tested. Despite the sample size varied across the models, the results of path a were nearly identical.  ^c^ Based on bias-corrected bootstrap with 5000 bootstrap samples.  ^d^ Path c and path c' were analyzed based on binary logistic regressions, and the coefficients were presented as a log-odds metric.  ^e^ The number of geriatric syndromes was counted from the presence of malnutrition risk, possible sarcopenia, frailty, falls, visual impairment, and hearing impairment. | | | | | | | | | | | | | | | | | | | | | | | | | | | |
